# Salary, flexibility or career opportunity? A choice experiment on gender specific job preferences

**DOI:** 10.3389/fsoc.2023.1154324

**Published:** 2023-04-17

**Authors:** Madlaina Jost, Sara Möser

**Affiliations:** ^1^Department of Sociology of Education, University of Bern, Bern, Switzerland; ^2^School of Social Work, Bern University of Applied Sciences, Bern, Switzerland

**Keywords:** preferences, gender roles, family formation, division of labor, gender inequality, discrete choice experiment, labor market, job choice

## Abstract

Using the evaluation of hypothetical job offers in a discrete choice experiment, we analyse which characteristics of employment positions are relevant to men and women when deciding between job offers. Thereby, we investigate whether preferences for work arrangements are gender specific. The analysis shows that on average, women have a stronger preference for part-time work than men, and that the career prospect of a job is more important to men than to women. Furthermore, we use heterogeneity within genders to study whether gender specific preference patterns result from gendered considerations of family formation. We find that certain men and women, especially those who plan to have children and have traditional intentions about the division of labor in the household, evaluate work relationships more strongly according to gender roles than others. This analysis of hypothetical employment choices provides valuable insight into the preference structure of men and women, which proves to be heterogeneous within and between genders.

## 1. Introduction

Women in Switzerland earn 19% less than men (Federal Statistical Office, 2021, p. 32), with consequences for their financial security in retirement and their economic independence (Madero-Cabib and Fasang, 2016). A large part of this gender pay gap is explained by chosen profession (Schmid, 2016). But even within occupations, women earn less than their male colleagues as they are more likely to work part-time (Federal Statistical Office, 2021, p. 41), more likely to take time off for care work (Federal Statistical Office, 2021, p. 20) and less likely to hold management positions (Federal Statistical Office, 2021, p. 38). Additionally, unpaid labor is unequally distributed among men and women, with women spending more time on care work, housework and volunteer work. Overall, women perform 60% of unpaid work, while men account for 61% of paid work (Federal Statistical Office, 2022).

The common explanation is that these gender specific employment situations within and across occupations and the resulting gender pay gap arise from different preferences (Hakim, 2002; Schmid, 2016). While gender preferences might be partly due to biological differences (Eagly and Wood, 2012), a large part are based on gender specific needs, primarily for reconciling work and family life (Polachek, 1981; Becker, 1985), which are said to result from the gendered division of labor, in which women are traditionally the primary caregivers and men the primary breadwinners of the family. Due to the persistence of this seemingly traditional division of labor in gender norms and stereotypical life course expectations, men and women develop different work preferences. Women value work arrangements that allow them to combine work with care responsibilities, e.g., good working hours, while men value high earnings in order to provide for the family.

The purpose of this study is to test this hypothesis of gender differences in job preferences by analyzing the evaluation of hypothetical job offers by childless men and women in their mid-twenties in a discrete choice experiment. Our contribution to the literature is threefold: first, by analyzing the extent to which specific job characteristics are taken into account when deciding between two job offers, we investigate whether the different work realities of men and women actually represent gendered preferences. Second, the analysis of preference heterogeneity between and within genders allows us to compare men and women with different intentions to start a family and attitudes toward the division of labor in the household. This is relevant to the question of whether gender specific preference patterns are actually the result of gender specific considerations about family formation. Third, much of the evidence on gender differences in work arrangements is based on either observational data on the de facto different situation in the labor market or on stated preference data. The use of choice experiments can be considered to be the most reliable method to reveal preference heterogeneity, because, unlike observational studies, they can control for selection effects and problems associated with endogeneity. Moreover, compared to stated preference, this method disentangles the effects of specific job characteristics, allowing us to identify their relative importance.

Our contribution provides valuable insights into preference heterogeneity within and between genders thereby extending existing knowledge on gendered patterns of employment arrangements. Hierarchical linear probability models of the choice experiment confirm that women have a stronger preference for part-time work than men, while career advancement is more important to men than to women. On closer inspection, these gender preference patterns are most pronounced among respondents who intend to have children and who have traditional attitudes toward the division of labor.

The article is structured as follows: First, the theoretical background of gender preferences is discussed, followed by a brief overview of the current state of research. This is followed by a discussion of the experimental design and the analysis strategy. The results are presented in three steps before finally, the limitations and need for further research are discussed, followed by the conclusions.

## 2. Gender specific preferences

Paid and unpaid labor is highly gendered regarding multiple dimensions. Both human capital theory (Becker, 1985) and social role theory (Eagly and Wood, 2012) trace the gender differences in the labor market back to the domestic specialization of work in families. The social role theory emphasizes the relevance of gender roles and stereotypes. Gender role expectations are embedded in the minds of individuals and shared with the community, resulting in a social consensus that forms the basis of social structures and culture (Eagly and Wood, 2012). In the process of socialization, men and women internalize specific gender roles and gender specific values (Eccles, 2011). While masculinity is associated with achievement, dominance and competition, femininity is described as nurturing and considerate (Williams and Best, 1990; Croson and Gneezy, 2009; Eagly and Wood, 2012). Following these gender stereotypes, men are more career focused, value professional advancement and are less likely to shy away from a competitive work atmosphere (Konrad et al., 2000), while women value a pleasant, collegial working environment (Williams and Best, 1990) and are less career-driven (Konrad et al., 2000). People live up to these expectations regarding the suitability of men and women for different tasks, thereby reinforcing gender roles (Eagly and Wood, 2012), because they believe that others will respond to them in a better way if they confirm their ascribed gender role, while deviant behavior will be punished (Anderson et al., 2001; Byron, 2007).

Women are expected to be responsible for housework and taking care of children, while men are expected to serve as the main breadwinner of a family (Polachek, 2004). Human capital theory (Becker, 1985) emphasizes that these gender roles affect women's and men's career choices and educational decisions differently. Women choose careers and work arrangements that are compatible with family responsibilities. Men, on the other hand, choose careers and jobs that allow them to meet the demands of being the primary breadwinner (Gabay-Egozi et al., 2014). This is driven by rational calculations leading men and women to deliberately prioritize either success or compatibility, and to invest in their human capital accordingly, depending on how they individually anticipate future family responsibilities. In other words, in line with the domestic division of labor, men invest more in the labor market than women, who invest more in the private sphere of family life, which consequently translates into higher wages and steeper careers for men. It is a self-perpetuating circle. When women work on average fewer hours than men, they gain less work experience and are thus confronted with fewer opportunities for advancement and lower wages by employers (Polachek, 2004).

The gender differences in social roles and investment in human capital lead, on the one hand, to men and women developing gendered occupational aspirations and choosing different professions (Polachek, 1981; Gottfredson, 2002). The Swiss labor market is characterized by large and persistent horizontal occupational gender segregation (Sousa-Poza, 2003; Becker and Glauser, 2015). On the other hand, working arrangements are gendered both within and between occupations: Women are paid lower salaries, are more likely to work part-time (Federal Statistical Office, 2021, p. 41) and are less likely to be in high level positions (Federal Statistical Office, 2021, p. 38). While horizontal occupational segregation and gendered working arrangements are interrelated aspects of gender differences in labor market participation, in our analysis we focus on this second aspect, the gender specific work preferences and investigate the differences in men and women's valuation of specific job characteristics.

In summary, social role theory and human capital theory show that the traditional division of roles between men and women emerges from the domestic division of labor, which in turn leads to gender differences in labor market investment and preferences for work arrangements. This theoretical background constitutes the basis for our main hypothesis that men and women have different preference structures with regard to work arrangements (Hypothesis 1). In line with gender roles and the traditional division of labor, we expect that women have a stronger preference for part-time work than men (Hypothesis 1a) and that the career prospects of a job are more important for men than for women (Hypothesis 1b). According to theory, these gender specific preference patterns are determined by gender specific considerations of family formation and career expectations. Therefore, we assume gender specific preferences to be more prevalent among men and women with family formation intentions (Hypothesis 2) and traditional gender role attitudes (Hypothesis 3).

## 3. State of research

Overall, the state of research confirms the theoretical assumption that men and women have different preferences for work arrangements. This is especially evident with regard to attributes that increase the family compatibility of a job (Konrad et al., 2000; Kaufman and White, 2015; Wiswall and Zafar, 2018; Petrongolo and Ronchi, 2020; Le Barbanchon et al., 2021).

While much of the state of research is based on measures of stated preferences, choice experiments are increasingly being conducted to study work-related preferences. Some interesting results have emerged from choice experiments on gendered job preferences, which we present below.

Wiswall and Zafar (2018) find clear gender differences in preferences for part-time work. According to their choice experiment, women are willing to give up over 7% of their salaries to work part-time, while men are only willing to give up just over 1%. Other research, however, concludes that while men are reluctant to work part-time and prefer full-time jobs (De Schouwer and Kesternich, 2022), women do not have a preference for part-time work, but consider part-time and full-time jobs to be equally attractive (De Schouwer and Kesternich, 2022; Non et al., 2022). The experimental evidence also shows that women value flexibility in the form of autonomy over working hours or the possibility of home office more than men do (Datta, 2019; Valet et al., 2021; De Schouwer and Kesternich, 2022; Non et al., 2022). According to Valet et al. (2021), it is more important for women to have a flexible job instead of a high-paying one, whereas for men these two characteristics are of equal importance. However, other studies do not find any gender difference in the evaluation of flexible working hours, home office possibility or overtime work (Datta, 2019; Seehuus, 2023). While several studies confirm the theoretical assumption that men place more value on higher wages due to the male breadwinner gender role (Wiswall and Zafar, 2018; Valet et al., 2021; Seehuus, 2023), other findings find no difference in the importance of wages between men and women (Non et al., 2022). Further differences are found in the preference for fixed wages over performance-based wages (Non et al., 2022), which is explained by varying degrees of competitiveness. Regarding the social impact of their employment, De Schouwer and Kesternich (2022) show that women consider it to be more important that their work has a positive effect on society. Moreover, women prefer working in the non-profit sector more than men (Non et al., 2022). Regarding the question of whether job security is more important to women or men, the experimental research literature has produced inconsistent results (Wiswall and Zafar, 2018; Datta, 2019; Valet et al., 2021; Non et al., 2022). Furthermore, the research literature also does not show clear gender differences in preferences concerning the reputation of the company, the gender composition of the workforce and the support of further training (Wiswall and Zafar, 2018; Valet et al., 2021).

With regard to the role of family formation and future division of labor, theory suggests that gendered preferences arise from different expected gender roles in families (Becker, 1985; Eccles, 2011). Some researchers confirm that family responsibilities are important in predicting gendered preferences. Childless women place a lower value on flexibility than mothers, while income and professional advancement are more important to fathers than to childless men (Corrigall and Konrad, 2006). Examining the role of attitudes toward the division of labor in mediating job preferences, there are pronounced differences within the groups of men and women. Using stated preference data from Sweden, Kaufman and White (2015) show that professional advancement is more important for egalitarian women, while traditional women especially value family-friendly job attributes such as part-time work. Additionally, egalitarian men prefer part-time work more strongly than traditional men. While Traditional men, on the other hand, are more concerned with wage and professional advancement. To date, no experimental research has analyzed family formation intentions and attitudes toward the division of labor as mediators of gender specific job preferences.

## 4. Data and methods

###  4.1. Data and description of the sample

In this contribution, we aim to test the hypotheses outlined in the previous section using the evaluation of hypothetical job offers in a discrete choice experiment (Louviere et al., 2000), that was included in the tenth wave of the DAB panel study (Becker et al., 2020). The DAB (Determinants of educational choices and vocational training opportunities) panel study tracks the educational and occupational trajectories of adolescents born around 1997 who concluded lower secondary education in regular classes of public schools in the German-speaking cantons of Switzerland in the summer of 2013. It is based on a stratified random sample of 8th grades of the 2011/12 school year that have so far been surveyed ten times. All respondents of the tenth survey wave participated in the survey experiment.^1^

At the time of the 10th wave, the sample of the DAB study (Nt10 1,829) was in their mid-twenties and is briefly described in the following section along relevant characteristics. While the majority (98%) has not yet started a family, two-thirds of respondents are in a committed relationship with a quarter living in a shared household with their partner. The majority of those surveyed (76%) state that they would like to have children at some point in their life and 15% of respondents say they do not. While half of all women want to have children in the next five years, only 35% of men say the same. When it comes to organizing care and work responsibilities within the family, more than half of the respondents have a balanced view, agreeing that women and men should share paid work and housework equally. A further 27% have traditional attitudes, regarding men as primary breadwinners and women as primary caregivers. Only 3% of respondents state anti-traditional attitudes, seeking a role reversal between typical masculine and feminine responsibilities. Almost all DAB respondents have completed secondary education at the time of the survey experiment. About a third had also completed some type of higher education. This proportion is higher for women. Furthermore, one third (28%) of the sample is currently enrolled in tertiary education. However, most participants (65%) are currently in paid employment.^2^ These proportions are balanced between genders. The detailed distribution of respondents' highest educational attainment, current employment, desire to have children and attitudes toward the division of labor is shown in Table S1 in the Appendix. The associations between current employment and intentions to start a family and gender roles are presented and discussed in the Appendix (Tables S2–S5).

###  4.2. Experimental design

In survey-based choice experiments, respondents are asked to choose the most preferred option among several alternatives (Louviere et al., 2000). By systematically varying the specific characteristics of the alternatives, it is possible to determine how important these characteristics are to the decisions under study. The use of choice experiments to measure preferences allows the analysis of counterfactuals in an environment of reduced complexity and complete transparency, thus providing the opportunity to single out the valuation of particular attributes for sociological research questions. The use of survey experiments and discrete choice experiments in particular is fairly novel in social science research (Auspurg and Liebe, 2011; Liebe and Meyerhoff, 2021), having originated in market and transport research where consumer preferences for products and services are of practical interest (Louviere et al., 2000; Liebe et al., 2021). In this paper, we apply a discrete choice experiment to investigate gendered preferences for working arrangements.

The respondents of the tenth survey wave of the DAB panel sturdy were asked to imagine that they are looking for a new job and have applied for various positions in their occupational field. They were presented with a series of hypothetical but realistic job offers, all within reasonable commuting distance and in line with their qualification. The positions varied systematically for a set of seven attributes representing cost and utility dimensions, which are shown in Table 1. The monetary compensation of work is taken into consideration by including monthly wage in relation to the average pay in the prospective industry (10% higher; 10% lower or as high as usual). Two attributes concerning the number of hours worked are included as measures of the compatibility of family and care obligations and employment. One describes the position's initial employment percentage (80 or 100%) and the other whether a future reduction is possible. Another attribute indicates whether the working hours are fixed, i.e. whether the employer dictates when one has to work or whether one can arrange one's working hours flexibly. The career potential of a job is described in the choice experiment by two attributes. On the one hand, whether the employer offers financial support for further training and whether it is possible to use working time for such training. On the other hand, by whether the job promises good career opportunities, i.e., a higher position in the company is in prospect. And finally, the working atmosphere is described as either more competitive or more collegial to characterize the interpersonal work environment.

**Table 1:** Example of a choice set.

**Attribute**	**Levels**
Wage	10% lower than usual or
	**as usual in the industry** or
	10% higher than usual
Workload	**100%** or 80%
Reduction of workload is	**not possible** or possible
Working hours are	**not flexible** or flexible
Company supports further training	**no** or yes
Opportunity for professional advancement	**no** or yes
Working atmosphere	**rather competitive** or rather collegial

Reference category is indicated in bold in the table above.

The hypothetical employment offers were presented in four choice sets of two alternatives each. Meaning that all participants of the tenth survey wave were asked four times to evaluate two hypothetical job offers that systematically differed in the characteristics described above and were displayed as a table. To generate these choice sets, we took a fractional factorial of 48 two-alternatives choice sets from the 192 possible combinations of the seven characteristics and group these in 12 blocks. The participants were assigned randomly to one of the 12 blocks. The D-efficiency is 93 when all two-way interactions are taken into account. For each choice set the respondents were asked to indicate which job offer of the two they find more attractive (forced choice) and which job offer they would prefer to accept if they also had the option of rejecting both (opt-out/unforced choice). One thousand eight hundred twenty-nine respondents took part and 7,163 preferences as well as 7,273 choices were collected. An illustration of the instructions to the respondents and one example choice set is provided in the appendix.

###  4.3. Analytical strategy and explanatory variables

To test our theoretical hypotheses regarding gendered preferences for work arrangements, we estimate the effect of an employment characteristic on the probability of choosing one position over the otherwise identical other using linear probability models nested in respondents and choice sets. This strategy allows considering the panel structure of the data (Auspurg and Liebe, 2011) and has statistical benefits compared to logistic regression (Mood, 2010; Gomila, 2020). In a first step we fit a linear probability model of all job attributes on the preference variable and include interaction terms of each job attribute with gender to analyse the gender difference in the valuation of each characteristic. The different preferences of men and women and the difference between them are shown separately for each model. In a second step, we estimate models for separate groups by family formation intention and expectations regarding the division of labor in families. The interaction terms between gender and each job attribute is included, allowing us to observe between-gender differences within these subgroups and test hypothesis 2 and 3. In a third step, a three-way interaction model is calculated with interaction terms between occupational characteristics, gender and the explanatory grouping variable, i.e., family formation intentions or attitudes toward division of labor. Based on this model, the estimated probabilities for men and women by subgroup are calculated and the differences between the estimates are tested, showing the different effects by subgroup within gender in a comprehensive way.

Data from the tenth survey wave of the DAB panel study is used to examine the explanatory power of family formation expectations and gender roles regarding gendered preferences for the division of labor in families. The respondents are divided into four groups by their answer to the questions “Do you intend to have children?” and “At what age can you imagine having your first child?”. The first group includes those who anticipate early parenthood and definitely want to have children in the near future (before the age of 30); the second group includes those who anticipate late parenthood and want to have children but not until after the age of 30; while those who anticipate childlessness, who probably or definitely do not want to have children, make up the third group. The fourth group contains those who did not answer this question or had not yet thought about having children. The results of this residual group are not further discussed as they are not of substantive interest. The calculation can be found in the Appendix (Table S8). Respondents were also asked what they thought is the best arrangement for organizing family and work life as a couple when they have young children. The six response options included two solutions corresponding to the conservative male breadwinner model (woman part-time or not working, man full-time), two egalitarian arrangements (both working full-time or part-time) and two anti-traditional arrangements (man part-time or not working, woman full-time). The estimation for those who did not answer this question are included in the Appendix (Table S9).

In this publication, we present results based on the choice of the respondents to the forced choice answer. There is an ongoing debate regarding the inclusion of an opt-out option in choice experiments. Forced choice can introduce bias by forcing respondents to choose between two offers, both of which they may find unsatisfactory (Campbell and Erdem, 2019). The inclusion of an opt-out option, however, more closely resembles a genuine job search process and allows the analysis of status quo effects (Meyerhoff and Liebe, 2009). Although in the experimental condition respondents were asked to imagine that they were currently looking for a new job, their status quo is not specified, so it is unclear whether rejecting both offers would result in hypothetical unemployment, staying in the current job, or some other situation. This can lead to bias in the effects due to the diversity of the respondents' initial circumstances. Additionally, the opt-out option can lead to loss in efficiency, as it is cognitively less challenging for the participants to refuse to choose, regardless the composition of the job offers (Veldwijk et al., 2014). A two-stage questioning process, as was implemented in the choice experiment analyzed in this contribution, does not demand much more effort from participants and allows controlling the robustness of the decisions (Scott and Witt, 2020). For the purpose of the following presentation of the results, the analyses have been calculated using the forced-choice response, indicating which of two job offers is considered more attractive. All analyses were replicated using the unforced opt-out response with only minor substantive differences, which are contrasted in the discussion section.

## 5. Results

###  5.1. Overall

**Figure 1:**
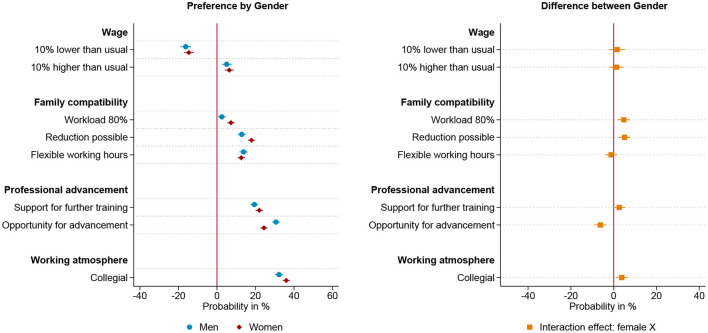
Results for all respondents based on linear probability model (*N* = 14,326) nested in respondents (*n* = 1,796) and choice sets (*n* = 7,193). **(Left)** Estimated change in probability by gender to choose the job offer given the specific level of the attribute. **(Right)** Interaction effect between gender and attribute. 95% confidence interval. Based on Table S7 in appendix.

Figure 1 shows the results of the choice experiment for all respondents. Each attribute's effect size reflects the change in probability of choosing the alternative, holding other attributes constant. All monetary and non-monetary job characteristics included influence the choice of employment position, as can be seen on the left-hand side of Figure 1. A higher income, flexible and reduced working hours, as well as professional advancement and further training opportunities increase the attractiveness of an offer. However, whether the hypothetical work environment is described as collegial rather than competitive is the most important characteristic for both men and women, followed by opportunities for advancement.

In line with hypothesis 1, there are gender differences in preferences for workload, possibility of reducing workload, career advancement opportunities and working atmosphere, as can be seen from the interaction effect on the right-hand side of Figure 1. Thereby, the direction of these effects is in line with theoretical expectations and the state of research: Women value a lower workload and a collegial working environment more highly than men do (hypothesis 1a), while men value career prospects to a greater extent than women do (hypothesis 1b). There are no gender differences concerning the valuation of salary, flexible working hours and the support of further training. While the gender differences are in line with theoretical expectations, women still value the opportunity for advancement more highly than the attributes that describe compatibility with family.

###  5.2. Family formation

The division of labor between men and women is at the center of the theory of gender differences in preferences. We examine preferences according to family formation plans in order to test whether hypothesis 2 holds, i.e., that women act in anticipation of the role of primary caregiver and men in anticipation of the role of primary breadwinner.

**Figure 2:**
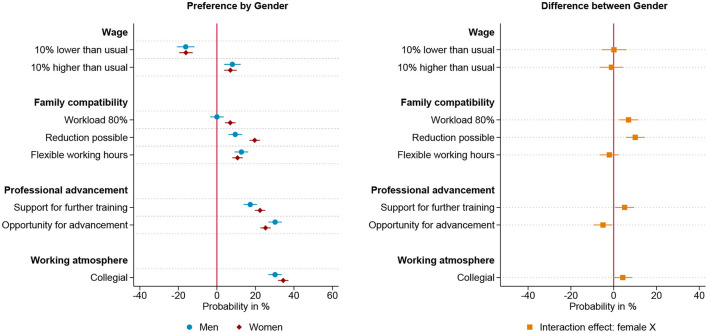
Results for intended early parents based on linear probability model (*N* = 6,134) nested in respondents (*n* = 767) and choice sets (*n* = 3,067). **(Left)** Estimated change in probability by gender to choose the job offer given the specific level of the attribute. **(Right)** Interaction effect between gender and attribute. 95% confidence interval. Based on Table S8 in Appendix.

**Figure 3:**
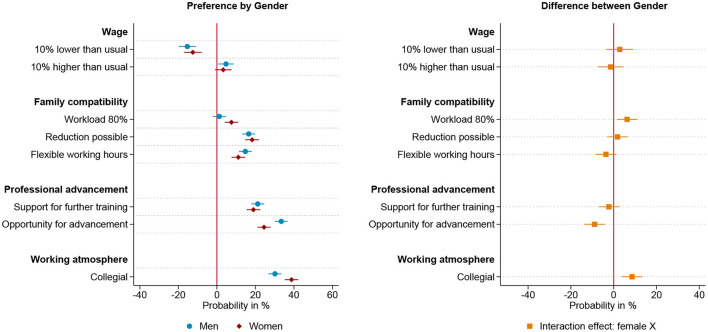
Results for intended late parents based on linear probability model (*N* = 4,698) nested in respondents (*n* = 588) and choice sets (*n* = 2,349). **(Left)** Estimated change in probability by gender to choose the job offer given the specific level of the attribute. **(Right)** Interaction effect between gender and attribute. 95% confidence interval. Based on Table S8 in Appendix.

**Figure 4:**
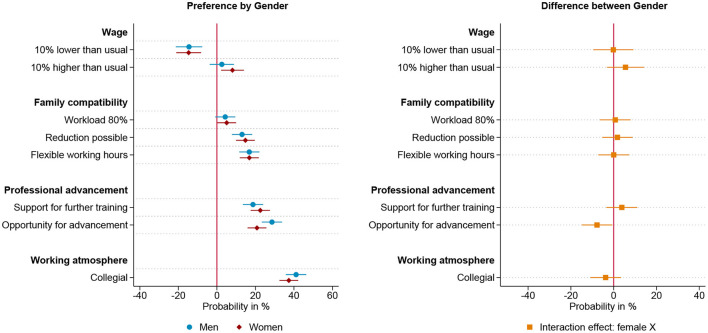
Results for intended childless respondents based on linear probability model (*N* = 2,130) nested in respondents (*n* = 267) and choice sets (*n* = 1,065). **(Left)** Estimated change in probability by gender to choose the job offer given the specific level of the attribute. **(Right)** Interaction effect between gender and attribute. 95% confidence interval. Based on Table S8 in Appendix.

Figures 2–4 present linear probability models of gendered preferences for working arrangements for three groups, those who anticipate to be parents before the age of 30, those who want children later and those who intend to stay childless. The preference structure of those respondents intending to start a family before the age of 30, meaning within the next five years, is plotted in Figure 2 and is similar to the overall model. While men who anticipate young fatherhood show no preference for part-time jobs, women who anticipate young motherhood prefer part-time jobs. Additionally, being able to reduce working hours has a significantly larger impact on the job choice of these women than men. Overall, however, for both men and women who want to have children early, a collegial working atmosphere and career advancement opportunities are more decisive than family-friendly working hours. Thereby, women show a slightly higher valuation of support for further training measures, while men show a slightly more pronounced emphasis on career advancement opportunities. In the group of respondents who intend to become parents after the age of 30, we find less pronounced gender specific differences in preferences, as shown in Figure 3. Both genders show an equally strong preference to reduce working hours and for further training opportunities. However, also in this group, the career perspective of the offered position is more decisive in men's job choice, while women show a stronger preference for part-time work. Moreover, the working environment is a more decisive factor for the women in this group than for the men. For men and women with the intention of starting a family beyond the age of 30, career advancement opportunities and collegial working atmosphere are the two most influential workplace characteristics, with the former being more influential for men and the latter more influential for women. Respondents who do not indent to become parents show hardly any gender differences in their preferences for work arrangements, these results are plotted in Figure 4. Women and men who do not want to have children both show no preference for part-time positions, and equally value flexible working hours and the possibility of reducing workload. Wage, training opportunities and the working atmosphere are also equally influential in the choice of positions for men and women in this group. However, even in this group of those anticipating childlessness, career prospect is a more decisive factor in men's choice of position than it is for women. In summary, the theoretically expected gendered preference pattern regarding part-time employment is found among respondents anticipating parenthood—not, however, among those respondents who intend to stay childless. The pattern that the career prospect of a position is more decisive for men than for women is found in all three groups studied, i.e. seemingly independent of the intention to start a family. Furthermore, the compensation of a position is equally important to men and women across different family formation scenarios.

###  5.3. Gender role attitudes

**Figure 5:**
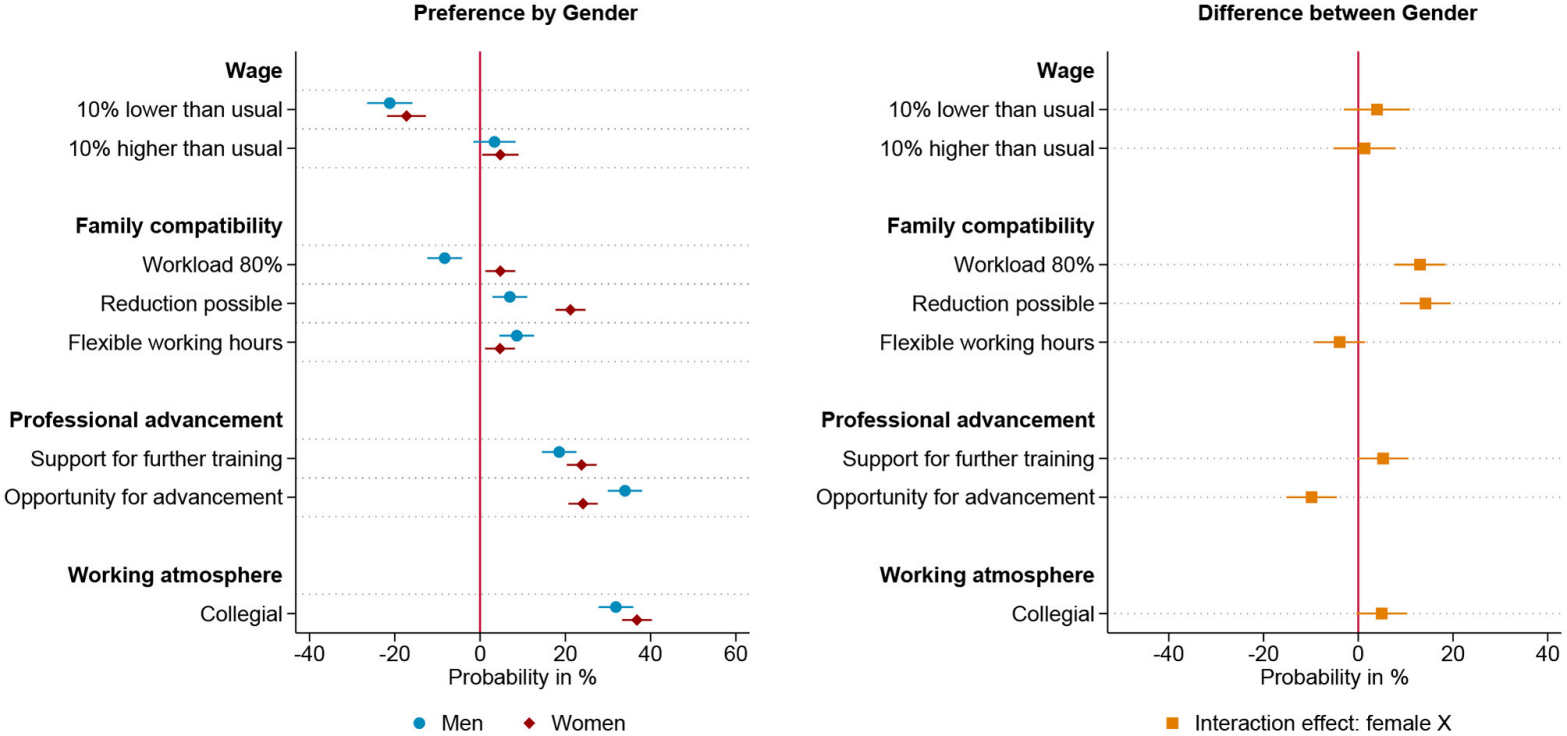
Results for traditional respondents based on linear probability model (*N* = 3,914) nested in respondents (*n* = 490) and choice sets (*n* = 1,957). **(Left)** Estimated change in probability by gender to choose the job offer given the specific level of the attribute. **(Right)** Interaction effect between gender and attribute. 95% confidence interval. Based on Table S9 in Appendix.

**Figure 6:**
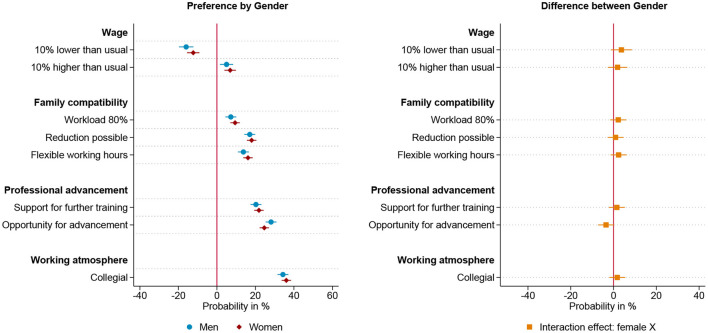
Results for egalitarian respondents based on linear probability model (*N* = 7,826) nested in respondents (*n* = 979) and choice sets (*n* = 3,913). **(Left)** Estimated change in probability by gender to choose the job offer given the specific level of the attribute. **(Right)** Interaction effect between gender and attribute. 95% confidence interval. Based on Table S9 in Appendix.

**Figure 7:**
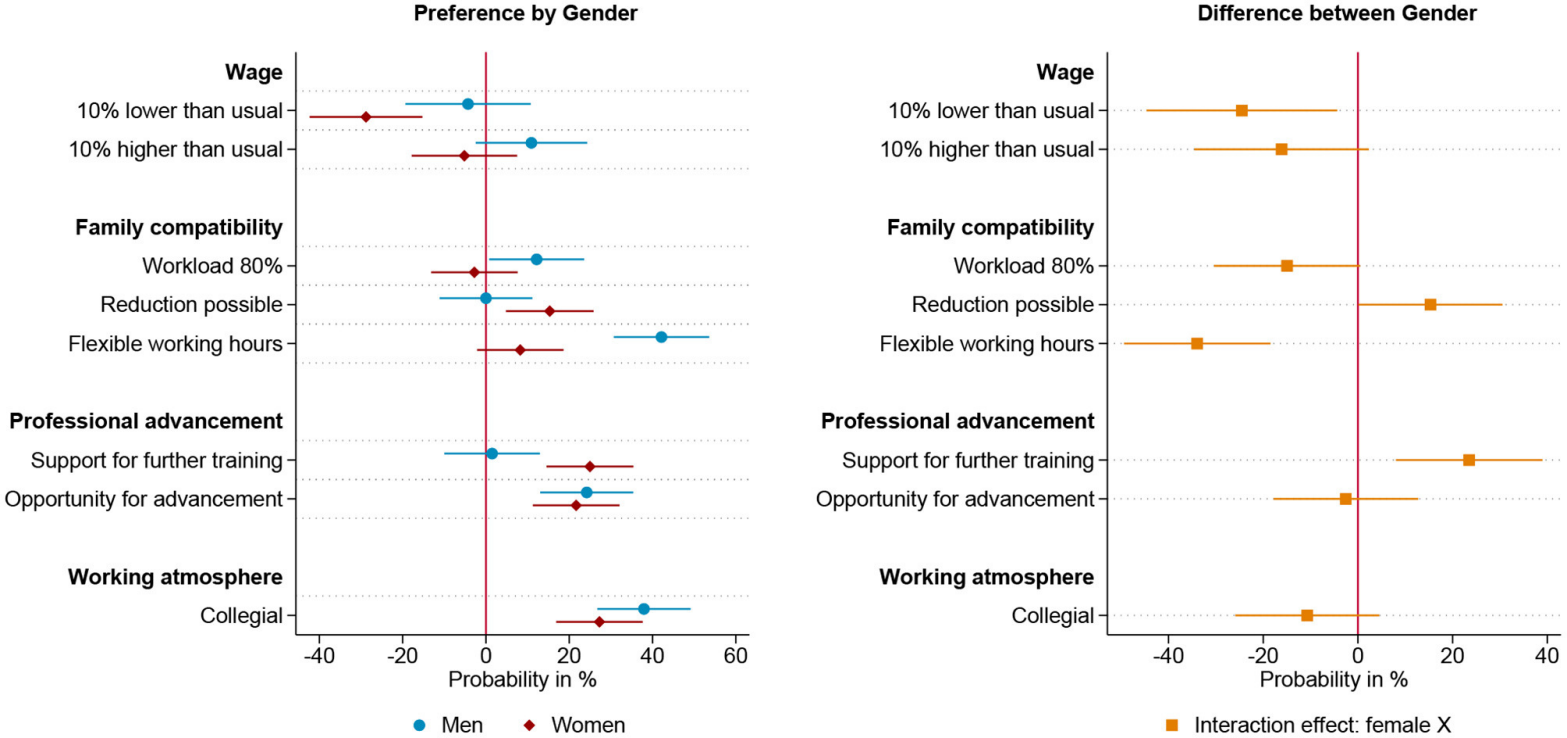
Results for anti-traditional respondents based on linear probability model (*N* = 490) nested in respondents (*n* = 61) and choice sets (*n* = 245). **(Left)** Estimated change in probability by gender to choose the job offer given the specific level of the attribute. **(Right)** Interaction effect between gender and attribute. 95% confidence interval. Based on Table S9 in Appendix.

To test the third hypothesis, that gender preferences are most prevalent among those who have internalized traditional their gender role attitudes, we divide our sample on the basis of their attitudes toward the division of labor in couples with young children. Among men and women who adhere to traditional gender role attitudes, i.e. male breadwinner and female caregiver, we find clear gender differences in the evaluation of work arrangements that corresponding to traditional gender roles, as shown in Figure 5. A collegial working atmosphere is the most influential workplace characteristic for traditionally oriented women, closely followed by the career prospects, support for further training and the possibility to reduce working hours, which factor into the decision to a similar extent. Traditional men, on the other hand, also rate the working atmosphere to be one of the most decisive factors next to career prospect. Remarkably, traditional men, show a negative preference for a part-time position and only a slight preference for the possibility to reduce the working hours. The only group that does not show any significant gender specific differences in the evaluation of job offers is the group of respondents who have an egalitarian attitude toward the division of labor in families, as shown in Figure 6. Figure 7 illustrates the results of the respondents with anti-traditional values toward the organization of paid and unpaid work in families. Even though this group is relatively small (61 respondents), there are remarkable and significant differences, regarding the evaluation of salary, flexible working hours and further training. Lower pay puts women with anti-traditional attitudes off, while it does not matter for anti-traditional men. Within this group, women demonstrate a preference for the reduction of working hours, while men prefer a part-time position. Remarkably, flexible working hours are one of the most important job characteristics for anti-traditionally minded men, while anti-traditionally minded women do not favor flexible working hours compared to fixed working hours. Whether the future company supports further training financially does not seem to matter for the decision of anti-traditional men, whereas it matters strongly for women. Additionally, both genders value opportunity for advancement and a collegial working atmosphere equally. These results are largely in line with reversed gender norms. Men who plan to be the primary caregiver value flexible working hours, while women who plan to be the primary breadwinner value further training and higher pay. Compared to men with anti-traditional values, women clearly reject low pay and part-time work, but nevertheless show no greater emphasis for advancement opportunities.

This subgroup analysis presented so far disentangles the overall results in respect to anticipated roles in the division of labor and yields the theoretically expected outcomes. Men and women with traditional values have different preferences for part-time work and career opportunities, with women expecting to be the family's primary carer and men the breadwinner. This gendered preference pattern is not found in egalitarian minded men and women, who intend to share paid and unpaid labor equally. Among men and women who endorse anti-traditional gender roles, women who would like to act as primary breadwinner and men as primary caregiver, marked differences in the valuation of work arrangements are found which are contrary to those in the traditional group.

###  5.4. Within-gender differences

As a third and final step, we present the results of two models that also compare within-gender differences: the first includes three-way interactions between job characteristics, gender and family intentions, and the second model includes interactions between job characteristics, gender and ideal work arrangements. Only the attributes that are of substantial interest are presented in the following.

**Figure 8:**
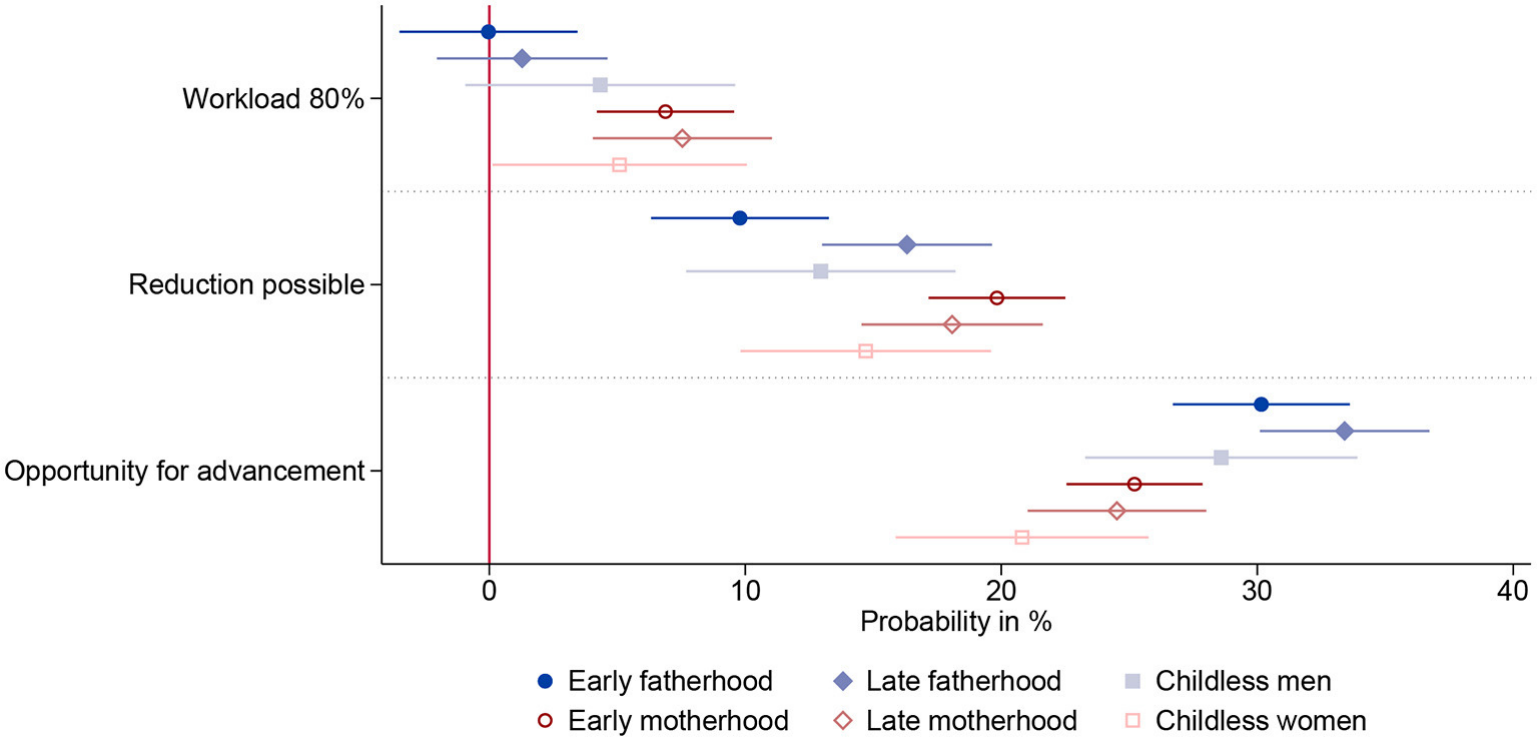
Results by family formation intentions based on linear probability model (*N* = 14,326) nested in respondents (*n* = 1,791) and choice sets (*n* = 7,163). Includes all three-way-interactions between attributes, gender and family formation intention. 95% confidence interval. Based on Table S10 in Appendix.

We observe some heterogeneity within gender when comparing men and women with different family formation intentions (Figure 8), however, only one within gender comparison is statistically significant. Men who do not expect to become fathers until after the age of 30 have a more pronounced desire to reduce their working hours than men who are planning to become fathers at an early age (χ^2^ = 7.14,  *p* < 0.01). So anticipating fatherhood does seem to influence how the option of reducing working hours is perceived. However, men do not show a statistically significant preference for part-time work, regardless of family formation intention, while women of all groups do. In summary, we find hardly any within gender differences regarding the evaluation of part-time work, the reduction of working hours and opportunity for advancement. However, as discussed in the previous analysis, the variation in preferences is large enough to detect substantial between gender differences in the respective subgroups of family formation intentions.

**Figure 9:**
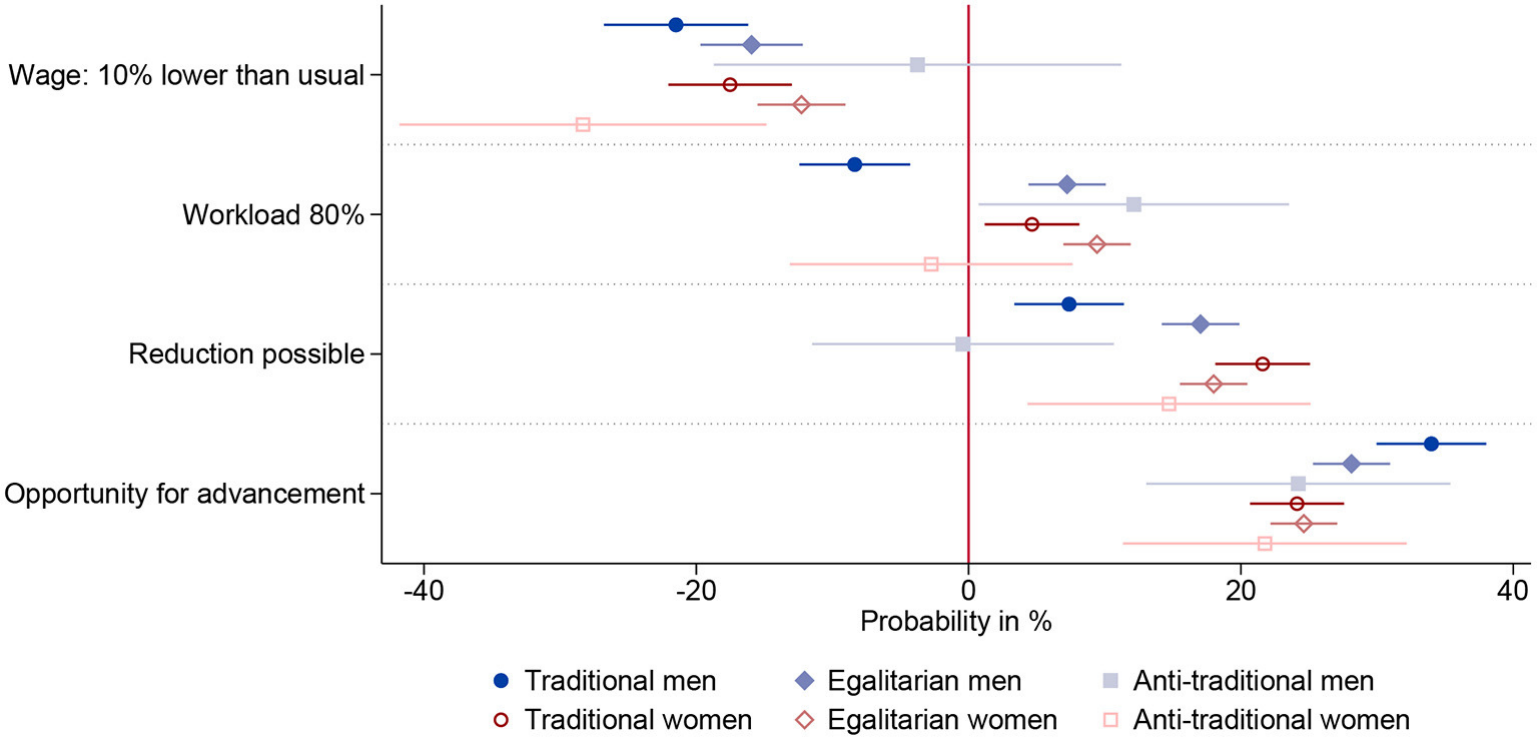
Results by attitudes toward division of labor based on linear probability model (*N* = 14,128) nested in respondents (*n* = 1,766) and choice sets (*n* = 7,064). Includes all three-way-interactions between attributes, gender and ideal division of labor. 95% confidence interval. Based on Table S11 in Appendix.

The pattern is more pronounced when combining the effects of gender and gender role attitudes, as presented in Figure 9. Comparing men and women with different attitudes to the division of paid and unpaid work, there are considerable differences in how much importance they attach to not being underpaid. Anti-traditional men are the only group who do not reject underpaid positions. They are less opposed to below-average pay than traditional men (χ^2^ = 4.80,  *p* < 0.05). Anti-traditional women, one the other hand, are more reluctant to accept a lower salary than egalitarian women (χ^2^ = 5.15,  *p* < 0.05). While all other subgroups prefer part-time positions over an otherwise equivalent full-time position, traditionally minded men prefer to work full time. On the other hand, anti-traditional women—who aspire to be the main breadwinner in a household with a partner who takes on the caring responsibilities—are indifferent between part-time and full-time offers. Thereby, women with egalitarian attitudes toward the division of labor show a stronger preference for part-time jobs than women with traditional (χ^2^ = 4.81, *p* < 0.05) or anti-traditional (χ^2^ = 5.00, *p* < 0.05) attitudes. Although anti-traditional men prefer part-time jobs, they do not favor the possibility of reducing working hours in the future. Egalitarian men want to be able to reduce their working hours much more than traditional (χ^2^ = 14.83,  *p* < 0.001) and anti-traditional men (χ^2^ = 8.94,  *p* < 0.01). While the group of anti-traditional minded men and women seem to be outliers regarding the evaluation of monetary compensation and compatibility, they demonstrate comparable preferences when it comes to opportunities for advancement. All groups of women desire professional advancement equally strong among each other (*p*>0.05 for all subgroups). Meanwhile, traditional men value advancement opportunities the most; significantly stronger than egalitarian men (χ^2^ = 5.45,  *p* < 0.05). Egalitarian men, on the other side, do not differ in their valuation of professional advancement compared to women, regardless of the latter's attitudes (*p*>0.05 for all subgroups).

In summary, both men and women choose their employment position based both on family compatibility and career prospect. The extent to which these criteria are taken into account in career choices varies by gender role attitudes. Differences are observed between egalitarian and traditional men, with egalitarians focusing on both compatibility and career prospects, whereas traditional men prioritize the latter. The pattern cannot be confirmed for women. While we do find differences in the evaluation of job attributes by gender role attitudes, hypothesis 3, that gender specific preferences are particularly pronounced among people with traditional attitudes, cannot be confirmed.

## 6. Discussion and conclusion

This paper analyses gender differences in preferences for different job attributes using a discrete choice experiment. According to gender role theory and human capital theory, men and women have internalized different values regarding their roles in paid and unpaid work. Women are socialized with the assumption that they will act as primary caregivers and therefore learn to value employment opportunities that allow them to balance work and family life, while men are socialized with the assumption that they will act as primary breadwinners and therefore come to emphasize high salaries and career advancement (Becker, 1985; Eagly and Wood, 2012). The 1,829 respondents of the DAB panel study (Becker et al., 2020) were presented four scenarios where they had to choose between two job offers which varied in the attributes of wage, workload, working hours, support for further training, opportunity for advancement and working atmosphere.

###  6.1. Main findings

For both men and women, opportunities for professional advancement and a collegial working environment are the most important job characteristics. Part-time positions, the possibility to reduce working hours and a collegial working atmosphere are more highly valued by women than by men, while men place more importance on opportunity for advancement than women. This confirms our first hypothesis that preferences for job characteristics differ between genders. To further investigate whether these gendered preference patterns are indeed the result of gendered considerations related to family formation and caring responsibilities, the heterogeneity of preferences between and within genders is compared, taking into account different intentions to start a family and attitudes toward the division of labor in the household. In general, the differences found are not conclusive enough to confirm hypotheses two and three. Irrespective of family formation intentions and attitudes, career prospects are more important for men than for women – with the exception of anti-traditional men, who do not differ significantly from women in this respect. However, the pattern of women preferring part-time positions and valuing the possibility of reducing their working hours in the future, while men seek full-time jobs, is particularly pronounced among those who expect to have children and among those with traditional values regarding the division of labor in the family. Among those who do not anticipate parenthood or have anti-traditional views, this pattern is not found or is even reversed. There are hardly any gender differences in terms of monetary compensation, support for training and flexibility of working hours, with the exception of anti-traditional men and women. While a collegial working atmosphere is one of the most important factors in the choice of workplace across all subgroups, gender differences are only found among those who expect to have children in the next few years.

In summary, our experiment confirms that preferences for work arrangements are gender specific. When deciding between job offers, different employment characteristics are of different importance for men and women. In particular, the unequal distribution of men and women in part-time and full time positions can be traced back to gendered preferences in line with the need for compatibility of work and family obligations. The fact that men are more likely to advance in their careers than women can also be attributed to their stronger emphasis on professional opportunities when choosing a new position. However, this pattern is not based on the need to be the family's main breadwinner or main carer, as men who do not intend to start a family also place this emphasis on career, while even women who do not expect to have childcare responsibilities are less career-oriented than their peers. Women do not seem to choose either family compatibility or career success at the expense of the other, as women do not differ in their assessment of career opportunities, regardless of intentions to start a family or ideal division of labor. Since the difference between men and women in terms of the importance of advancement opportunities persists across different individual situations, we conclude that this is deeply rooted in gendered role socialization based on stereotypical beliefs of masculinity and femininity. However, it is also important to note that even when gender differences are present, career advancement is one of the most important job characteristic for both men and women in the majority of subgroups.

###  6.2. Limitations

While our study adds to the research literature on gendered job preferences and provides insight into the mechanisms involved, some limitations regarding the experimental design should be mentioned. The vignettes include only a limited range of employment characteristics. Labor market research has shown that the employment situations of men and women vary on a huge range of dimensions (Federal Statistical Office, 2021), with pay, working hours and career advancement being only the most prominently discussed. Only seven attributes representing cost and utility dimensions were included in the design of this choice experiment. When choosing the levels and dimensions, the number of available respondents, as well as the precision of the estimates and the available estimation methods—in our case the need for subgroup analyses and interaction models—have to be taken into account (Auspurg and Hinz, 2011). Even with the limited set of dimensions, some of the subgroup analyses, especially regarding the small group of respondents with anti-traditional attitudes, produce results with high statistical uncertainty that must be interpreted with caution. There may have been more pronounced gender differences if an even larger sample had been available. Due to these considerations, the focus of this study is on attributes related to family compatibility and the career prospects of an employment position, with the addition of the attribute of work atmosphere. Future experimental analyses should, on the one hand, investigate these factors more closely by including a greater variation of these characteristics, i.e. not only part-time jobs with a workload of 80%, but also positions with lower workloads. On the other hand, further choice experiments should be conducted that include those characteristics that have been identified as being gender specific in the previous preference analysis but have not yet been the subject of experimental designs, e.g. the social impact or sustainability of a company. Furthermore, is has to be pointed out, that we cannot be sure that the wording of the attributes wad understood in the way we intended. In particular, the level of having flexible working hours did not produce the results we expected theoretically. This could be due to the fact that the participants had different ideas about the meaning of flexibility.

The experimental design used a two-stage questioning procedure, asking participants to indicate which position they find more attractive and which they would choose if they also had the option of rejecting both. The results presented in this paper are based on the response to the first question, where respondents were forced to choose between Offer A and Offer B. As a robustness check, all analyses were also replicated using the answer to the second question, with the option to Opt-Out. While overall and in most subgroups the results were substantially and statistically very similar, we find small differences regarding wage among people planning early fatherhood. In this subgroup, men are statistically significantly more likely than women to value higher wages. In addition, among non-traditional people, there is no difference between men and women regarding wage and flexible working hours when using unforced choice instead of forced choice. There is an ongoing debate on the inclusion of opt-out options in choice experiments and how these answers are to be interpreted (Meyerhoff and Liebe, 2009; Campbell and Erdem, 2019). While we are confident that our main findings are robust, further analysis of the effects of question wording and response options, as well as status quo effects, will be undertaken. However, to answer these methodological questions would have been beyond the scope of this paper.

Additionally, there are some limitations regarding the external validity of this experimental study. On the one hand, the method of choice experiments is hypothetical in three respects. First, the situation of choosing between two job offers is hypothetical. When looking for a job, applicants usually have a number of possible jobs in mind which are compared with each other, regardless of whether they are actually available to them, while in the best case they choose between several offers or are offered only one job. Second, the characteristics selected in the vignettes and their combinations do not necessarily correspond to the job offers that would be presented to respondents in the real world. The participants might work in certain occupations where it is highly unlikely to be offered flexible hours or work part-time. Additionally, there may be other characteristics that people consider when applying for a job that are not included in the attributes of the choice experiment, making the task of choosing between the described positions more unrealistic. As two thirds of our sample already entered the labor market, their preferences might also be influenced by restrictions they meet in their respective occupation. Therefore, their preferences might be influenced by their previous choices in the labor market and not vice versa, where they first form preferences and choose accordingly. Overall, it would be interesting to observe what type of jobs the participants have in five years and whether their choice then fits their preferences now. Third, the hypothetical nature of the method of choice experiments means that the decisions are without real-life consequences for the respondents. This can lead to hypothetical bias in response behavior, which is associated with a lack of external validity and can lead to biased results, particularly due to social desirability (Liebe et al., 2021). However, the indirect evaluation method of choice sets is considered more immune to such effects than direct queries of stated preferences (Louviere et al., 2000, p. 351) and, although hypothetical bias is an undeniable problem when interpreting results of choice experiments, the state of research on the subject is rather confident that survey experiments are reflective of actual real-world preferences and intentions (Haghani et al., 2021).

With regard to the generalisability of our results, it should be noted that the survey experiment on gendered preferences was implemented in the DAB panel study which tracks the educational and occupational trajectories of adolescents born around 1997 from the German-speaking part of Switzerland. Switzerland is a liberal-conservative country (Combet and Oesch, 2019), where traditional values regarding gender roles prevail (Oehrli et al., 2022) and the most common division of labor in families corresponds to the male breadwinner model (Lütolf and Stadelmann-Steffen, 2022). While the employment rate of women is much higher than 30 years ago, a majority of women, even young women, work part-time (Federal Statistical Office, 2020). Switzerland has one of the least affordable childcare systems worldwide, a short maternal leave and almost no paternal leave (Gromada and Richardson, 2021). Gender specific work patterns are influenced by conservative family policies and traditional gender norms in Switzerland and may differ from countries with more affordable care arrangements and longer parental leave (Gromada and Richardson, 2021; Oehrli et al., 2022). The survey experiment was conducted when the sample the DAB study was in their mid-twenties. All DAB participants who responded to the tenth survey wave were asked to imagine that they are looking for a new job and have applied for various positions in their occupational field. However, the current life situation of these respondents is heterogeneous, both in terms of educational level and employment situation, as well as in terms of their stage of life and expectations regarding partnership and family formation, as described in more detail in the description of the sample in the data section. The analysis presented in this paper has not been able to consider this heterogeneity adequately. Further analysis shows, that the extent and direction of gender differences in preferences for work arrangement vary depending on the employment situation and educational level (results provided in Table S12 in Appendix). For respondents currently employed with a qualification from secondary education gender differences are found regarding the family compatibility of a position as well as regarding advancement opportunities. While among those who are employed and have a degree from tertiary education gender differences are only found regarding the importance of part time work. Comparing men and women who are still in education we find that women value reduction possibilities to a greater extent than men do, while there are no gender differences regarding part-time work. Additionally, the preferences for flexible working hours are reversed, with men valuing flexible working hours more than women in education. This heterogeneity of current employment situations could not be adequately addressed in the main analysis presented in this article. Therefore our findings represent a necessary generalization of gender differences across different contexts. In addition, as more women than men in our sample have a tertiary level of education, it is possible that the interaction between gender and level of education may have had an impact on our overall results. Further analysis focusing on more specific subpopulations and the interrelatedness of life domains of employment and family formation^3^ would provide further valuable insights into gendered employment patterns. Regarding the generalisability of our research it should further be emphasized that the majority of respondents in our study do not have children. On the one hand, this allows us to analyse how the labor market decisions of young adults are influenced by the expectations of starting a family. We study young adults who are thinking about their future employment prospects at a stage in their lives before they have family responsibilities. On the other hand, focusing on one age cohort also limits the generalisability of our conclusion. We can not answer the question of how gender roles and work preferences differ between different age groups or how they develop over time. Follow-up research tracking the development and change of attitudes toward work arrangements over the life course would be an important addition to the literature.

## 7. Conclusion

The central question of this paper is whether women and men prefer different job characteristics and whether this is driven by gendered role expectations regarding the division of labor into paid employment and unpaid house- and care work. The results show that a collegial working environment and opportunities for career advancement are the most important factors for young adults when choosing a new employment position. In line with theoretical expectations derived from social role theory and human capital theory, there are pronounced gender differences in preferences for part-time work. Especially among those who want to have children and those with traditional attitudes, women prefer part-time work, while men prefer full-time work. This reflects the reality of the Swiss labor market, where women are significantly more likely than men to be working part-time. However, our experiment shows that men and women who do not plan to have a family or who have egalitarian or non-traditional attitudes do not conform to this pattern. A large proportion of male employees would be in favor of part-time work if it were available in their occupation. Furthermore, in almost all subgroups the pattern is that men place a higher value on career advancement than women. This is true regardless of whether the compared men and women want to have children and regardless of their attitudes to the division of labor. The fact that women are less likely to hold positions of responsibility thus corresponds to the argument that this is more important to men than to women, however, this cannot be attributed to gender role expectations in the family. Although less so than men, women also attach great importance to the career prospects of their new job. However, in comparison with men, they are less willing to prioritize it at the expense of other job characteristics, especially work-life compatibility. For women, career progression is one of many important factors taken into account when choosing between job offers, whereas for men, career perspective is by far the most important characteristic a new job has to fullfil, along with the working atmosphere. Moreover, it is just as important for women to have high wages as it is for men, regardless of their anticipated family situation. The gender pay gap cannot be attributed to women's willingness to forego pay in exchange for family-compatible working conditions.

Labor market participation and employment arrangements of men and women in Switzerland are highly differentiated by gender. Our experiment confirms that there are gender differences in young men's and women's preferences for work arrangements, such as part-time work and career prospects, which can be seen as a driving force behind gender differences in the labor market. However, the gender specific work realities cannot be attributed to differences in preferences alone. Men and women do have slightly different preferences for some employment characteristics. On the whole, however, men's and women's preferences for working arrangements are much less divided than the realities of the labor market. Furthermore, gender differences in the labor market are not only driven by gendered preferences; gendered preferences are also the result of socialization and deliberation, given the reality of occupational segregation. The unequal work realities faced by women and men—in terms of wage inequality, experiences of discrimination, career opportunities, availability of part-time and full-time jobs, etc.— influence their expectations of working life and are therefore reflected in young women's and men's employment preferences.

## Data availability statement

The raw data supporting the conclusions of this article will be made available by the authors, without undue reservation.

## Ethics statement

Ethical review and approval was not required for the study on human participants in accordance with the local legislation and institutional requirements. The patients/participants provided their written informed consent to participate in this study.

## Author contributions

MJ: concept, design, analysis, and writing. SM: concept, design, and writing. All authors have made a substantial, direct, and intellectual contribution to the work and approved it for publication.

## Footnotes

^1^More Information on the DAB panel study, as well as a detailed description of the sample selection and response rate, can be found on the website dab.edu.unibe.ch. The data sets of the first four waves are available at SWISSUbase. The experimental data can be requested from the authors.

^2^The assignment to the group of employees or students is based on their primary activity, i.e. the activity that the respondents considered to be most relevant and time-consuming. A third of respondents whose main activity at the time of the survey was paid employment participate in some form of training either as part of or in addition to their employment. Additionally, part-time employment while studying is common in our sample, with 48% of students having a student job.

^3^The interrelatedness of life domains of employment and family formation in our sample is discussed in the Appendix Tables S2–S5.
