# Supplementary material for: Salary, flexibility or career opportunity? A choice experiment on gender specific job preferences
Source: Front Sociol. 2023 Apr 17;8:1154324. doi: 10.3389/fsoc.2023.1154324 (PMC10150105; doi:10.3389/fsoc.2023.1154324)
Supplement: Supplementary file 1 [file Data_Sheet_1.pdf]

Supplementary Material

1 DESCRIPTIVE STATISTICS

Table S1. Descriptive Statistics

	Women		Men		Total	
	Frequency	Percent	Frequency	Percent	Frequency	Percent
Highest degree						
Primary education	44	4%	46	6%	90	5%
Secondary education	565	56%	598	72%	1163	64%
Tertiary education	395	39%	181	22%	576	31%
Occupational situation						
Working	660	66%	534	65%	1194	65%
In Education	275	27%	241	29%	516	28%
Other	69	7%	50	6%	119	7%
Family Formation Intention						
Early parenthood	492	49%	291	35%	783	43%
Late parenthood	282	28%	317	38%	599	33%
No children	146	15%	125	15%	271	15%
Not clear	84	8%	92	11%	176	10%
Ideal division of labour						
Traditional	288	29%	214	26%	502	27%
Egalitarian	570	57%	425	52%	995	54%
Anti-traditional	32	3%	31	4%	63	3%
Not clear	99	10%	144	17%	243	13%
N	1’004		825		1’829	

2 CROSS TABLES OF EXPLANATORY VARIABLES AND EMPLOYMENT SITUATION BY GENDER

Tables S2 - S5 present cross tables of family formation intention and attitudes towards division of labour with employment situation by gender. Overall, there are correlations between employment situation and the explanatory variables. Women are over-represented in the group of people in employment who have completed tertiary education, while more men are in employment after having completed only secondary education. For example, 37% of women who anticipate early motherhood are currently employed with tertiary education, while this proportion if only 16.15% for men anticipating early parenthood. In relation to the mean distribution of employment situations, employed men with tertiary education are overrepresented in the group of people who do not want children. Meanwhile, women in employment with tertiary education are underrepresented in the group of people who do not want children. Additionally, there are pronounced differences regarding the attitudes towards the division of labour. Traditional men and women are mainly employed with secondary education, while egalitarian people are mainly still in tertiary education. The proportions of people with egalitarian values across the different employment situations are similar to the mean distribution across the different employment situations as a whole. The group of people with anti-traditional values are mainly women in education and men who are in employment with secondary education. These correlations mainly show that family formation intentions and attitudes towards the

division of labour differ between employment and education context. While men and women are differently represented across the different employment contexts, the correlation between context and family formation / attitudes is however quite similar for both genders.

Table S2. Two-way table of family formation intention and current employment situation for female respondents

	Employed with secondary ed.	tertiary ed.	In education	Other	Total
Early parenthood (N = 492)	34%	37%	24%	5%	100%
Late parenthood (N = 282)	37%	24%	32%	7%	100%
No children (N = 146)	38%	22%	31%	9%	100%
Not clear (N = 84)	35%	26%	27%	12%	100%
Total (N = 1004)	36%	30%	27%	7%	100%

Table S3. Two-way table of family formation intention and current employment situation for male respondents

	Employed with secondary ed.	tertiary ed.	In education	Other	Total
Early parenthood (N = 291)	52%	16%	29%	3%	100%
Late parenthood (N = 317)	46%	10%	35%	9%	100%
No children (N = 125)	54%	18%	23%	6%	100%
Not clear (N = 92)	63%	12%	20%	5%	100%
Total (N = 825)	51%	14%	29%	6%	100%

Table S4. Two-way table of attitudes towards division of labour and current employment situation for female respondents

	Employed with secondary ed.	tertiary ed.	In education	Other	Total
Traditional (N = 288)	46%	31%	16%	7%	100%
Egalitarian (N = 570)	28%	32%	34%	6%	100%
Anti-traditional (N = 32)	19%	25%	47%	9%	100%
Not clear (N = 99)	53%	17%	21%	9%	100%
Total (N = 989)	36%	30%	28%	7%	100%

Table S5. Two-way table of attitudes towards division of labour and current employment situation for male respondents

	Employed with secondary ed.	tertiary ed.	In education	Other	Total
Traditional (N = 214)	63%	17%	17%	3%	100%
Egalitarian (N = 425)	42%	12%	38%	7%	100%
Anti-traditional (N = 31)	68%	10%	13%	10%	100%
Not clear (N = 144)	56%	13%	24%	7%	100%
Total (N = 814)	51%	14%	29%	6%	100%

3 EXAMPLE OF A CHOICE SET

Thank you very much for your answers so far. In the next part of the questionnaire, we are interested in how important different job characteristics are to you. Please imagine the following situation: You are looking for a new job and have applied for different job offers. You receive two job offers in your desired occupation, both of which have a similarly long commute, match your training and otherwise only differ in the following points:

- **Wage:** Whether the wage is higher, lower or the same as comparable positions in the industry with the same workload.
- **Workload:** Whether it is a full-time position (100%) or a part-time position (80%) and whether you have the option to reduce the workload or not.
- **Working hours:** Whether the working hours are fixed and determined by the employer or whether you can arrange your working hours flexibly.
- **Further training:** Whether or not your future employer is willing to contribute to your further training financially and whether or not you can devote working hours towards such training.
- **Professional advancement:** Whether or not the company offers you the possibility of career advancement, e.g. whether a more senior position in your field is likely to become available in the near future.
- **Working atmosphere:** Whether the working atmosphere can be described as being more competitive or more collegial.

In the following four situations, we would like to ask you to decide which job offer you find more attractive and which you would like to accept. You can also reject both offers.

Table S6. Example of a choice set

Attribute	Offer A	Offer B
Wage	usual in the industry	10% higher than usual
Workload	80%	100%
Reduction of workload is	possible	not possible
Working hours are	flexible	fixed
Company supports further training	yes	no
Opportunity for professional advancement	yes	yes
Working Atmosphere	rather competitive	rather collegial
Which job would you prefer?	Offer A	Offer B
	O	O
Which job do you choose?		
I reject both offers	Offer A	Offer B
O	O	O

4 LINEAR PROBABILITY MODELS

Table S7. Linear probability model of all participants

Wage: (Ref: as usual)		
10% lower	−0.162***	(0.014)
10% higher	0.050***	(0.013)
Workload: 80% (Ref: 100%)	0.025*	(0.011)
Reduction: possible (Ref: not possible)	0.129***	(0.011)
Working hours: flexible (Ref: fixed)	0.138***	(0.011)
Support for further training: yes (Ref: no)	0.194***	(0.011)
Opportunity for advancement: yes (Ref: no)	0.307***	(0.011)
Atmosphere: collegial (Ref: competitive)	0.322***	(0.011)
Alternative-specific constant: Option B	0.057***	(0.007)
Gender: female (Ref: male)	−0.052*	(0.021)
Interaction effect: female X		
Wage: 10% lower	0.016	(0.019)
Wage: 10% higher	0.013	(0.017)
Workload: 80%	0.047***	(0.014)
Reduction: possible	0.051***	(0.014)
Working hours: flexible	−0.012	(0.014)
Support for further training: yes	0.026	(0.014)
Opportunity for advancement: yes	−0.062***	(0.014)
Atmosphere: collegial	0.037**	(0.014)
Constant	−0.058***	(0.016)
lns1_1_1	−28.106***	(0.307)
lns2_1_1	−27.070***	(0.338)
lnsig_e	−0.870***	(0.006)
N (Choices)	14326	
N (Choice sets)	7193	
N (Respondents)	1796	

Linear probability model nested in participants and choice sets

Standard error in parentheses; * p<0.05, ** p<0.01, *** p<0.001

Table S8. Linear probability model by family formation intention

	Early parenthood		Late parenthood		No parenthood		Other	
Wage: (Ref: as usual)								
10% lower	−0.162***	(0.023)	−0.153***	(0.022)	−0.145***	(0.035)	−0.215***	(0.043)
10% higher	0.080***	(0.022)	0.047*	(0.021)	0.026	(0.032)	−0.011	(0.037)
Workload: 80% (Ref: 100%)	−0.000	(0.018)	0.013	(0.017)	0.043	(0.027)	0.127***	(0.031)
Reduction: possible (Ref: not possible)	0.095***	(0.018)	0.165***	(0.017)	0.131***	(0.027)	0.108***	(0.032)
Working hours: flexible (Ref: fixed)	0.127***	(0.018)	0.148***	(0.017)	0.168***	(0.027)	0.099**	(0.032)
Support for further training: yes (Ref: no)	0.173***	(0.018)	0.212***	(0.017)	0.187***	(0.027)	0.220***	(0.032)
Opportunity for advancement: yes (Ref: no)	0.302***	(0.018)	0.334***	(0.017)	0.286***	(0.027)	0.276***	(0.032)
Atmosphere: collegial (Ref: competitive)	0.302***	(0.018)	0.301***	(0.017)	0.411***	(0.027)	0.336***	(0.032)
Alternative-specific constant: Option B	0.045***	(0.011)	0.067***	(0.013)	0.063***	(0.019)	0.070**	(0.024)
Gender: female (Ref: male)	−0.093**	(0.033)	−0.011	(0.037)	0.007	(0.054)	−0.141*	(0.069)
Interaction effect: female X								
Wage: 10% lower	0.001	(0.029)	0.028	(0.032)	−0.002	(0.048)	0.081	(0.060)
Wage: 10% higher	−0.010	(0.028)	−0.014	(0.030)	0.055	(0.044)	0.108*	(0.055)
Workload: 80%	0.069**	(0.023)	0.063*	(0.025)	0.007	(0.036)	0.008	(0.045)
Reduction: possible	0.101***	(0.022)	0.018	(0.025)	0.017	(0.036)	0.016	(0.046)
Working hours: flexible	−0.020	(0.023)	−0.036	(0.025)	0.000	(0.037)	0.125**	(0.046)
Support for further training: yes	0.051*	(0.022)	−0.022	(0.025)	0.038	(0.037)	0.073	(0.046)
Opportunity for advancement: yes	−0.050*	(0.022)	−0.089***	(0.025)	−0.078*	(0.037)	−0.035	(0.046)
Atmosphere: collegial	0.042	(0.022)	0.086***	(0.025)	−0.038	(0.037)	−0.021	(0.045)
Constant	−0.002	(0.027)	−0.092***	(0.027)	−0.112**	(0.041)	−0.058	(0.050)
lns1_1_1	−29.159***	(0.413)	−28.426***	(0.600)	−28.811***	(0.789)	−28.321***	(0.873)
lns2_1_1	−29.128***	(0.484)	−27.407***	(0.543)	−27.549***	(0.835)	−29.574***	(0.957)
lnsig_e	−0.868***	(0.009)	−0.873***	(0.010)	−0.887***	(0.019)	−0.881***	(0.019)
N (Choices)	6134		4698		2130		1364	
N (Choice sets)	3067		2349		1065		682	
N (Respondents)	767		588		267		170	

Linear probability model nested in participants and choice sets

Standard error in parentheses; * p<0.05, ** p<0.01, *** p<0.001

Table S9. Linear probability model by gender role attitudes

	Traditional		Egalitarian		Anti-traditional		Other	
Wage: (Ref: as usual)								
10% lower	−0.212***	(0.027)	−0.160***	(0.019)	−0.043	(0.077)	−0.116***	(0.034)
10% higher	0.034	(0.025)	0.050**	(0.017)	0.109	(0.068)	0.067*	(0.031)
Workload: 80% (Ref: 100%)	−0.083***	(0.021)	0.072***	(0.014)	0.122*	(0.058)	0.053*	(0.026)
Reduction: possible (Ref: not possible)	0.070***	(0.021)	0.171***	(0.014)	0.000	(0.057)	0.109***	(0.026)
Working hours: flexible (Ref: fixed)	0.086***	(0.021)	0.138***	(0.015)	0.422***	(0.059)	0.193***	(0.026)
Support for further training: yes (Ref: no)	0.186***	(0.021)	0.203***	(0.014)	0.015	(0.059)	0.213***	(0.026)
Opportunity for advancement: yes (Ref: no)	0.340***	(0.021)	0.281***	(0.014)	0.242***	(0.057)	0.331***	(0.026)
Atmosphere: collegial (Ref: competitive)	0.319***	(0.021)	0.343***	(0.014)	0.379***	(0.057)	0.271***	(0.026)
Alternative-specific constant: Option B	0.040**	(0.014)	0.062***	(0.010)	0.088*	(0.039)	0.088***	(0.020)
Gender: female (Ref: male)	−0.137***	(0.040)	−0.044	(0.028)	0.262*	(0.113)	0.071	(0.063)
Interaction effect: female X								
Wage: 10% lower	0.039	(0.036)	0.037	(0.025)	−0.245*	(0.103)	−0.057	(0.053)
Wage: 10% higher	0.014	(0.033)	0.019	(0.023)	−0.161	(0.094)	0.018	(0.049)
Workload: 80%	0.130***	(0.027)	0.022	(0.019)	−0.150	(0.079)	−0.028	(0.041)
Reduction: possible	0.142***	(0.027)	0.010	(0.019)	0.153*	(0.078)	−0.005	(0.041)
Working hours: flexible	−0.039	(0.027)	0.024	(0.019)	−0.339***	(0.079)	−0.023	(0.041)
Support for further training: yes	0.052	(0.027)	0.016	(0.019)	0.235**	(0.079)	−0.075	(0.041)
Opportunity for advancement: yes	−0.098***	(0.027)	−0.035	(0.019)	−0.025	(0.078)	−0.083*	(0.041)
Atmosphere: collegial	0.049	(0.027)	0.017	(0.019)	−0.107	(0.078)	0.098*	(0.041)
Constant	0.075*	(0.032)	−0.107***	(0.022)	−0.168*	(0.082)	−0.123**	(0.041)
lns1_1_1	−20.476***	(0.527)	−24.304***	(0.455)	−27.482***	(1.699)	−28.552***	(0.911)
lns2_1_1	−21.323***	(0.619)	−24.851***	(0.452)	−28.368***	(1.439)	−28.754***	(0.876)
lnsig_e	−0.877***	(0.011)	−0.886***	(0.008)	−0.878***	(0.032)	−0.852***	(0.016)
N (Choices)	3914		7826		490		1898	
N (Choice sets)	1957		9813		245		949	
N (Respondents)	490		979		62		378	

Linear probability model nested in participants and choice sets

Standard error in parentheses; * p<0.05, ** p<0.01, *** p<0.001

Table S10: Linear probability model with three-way interaction: Intention for Family formation X Gender X Job Attribute

Wage: (Ref: as usual)		
10% lower	-0.164^{***}	(0.023)
10% higher	0.080^{***}	(0.022)
Workload: 80% (Ref: 100%)	-0.000	(0.018)
Reduction: possible (Ref: not possible)	0.098^{***}	(0.018)
Working hours: flexible (Ref: fixed)	0.128^{***}	(0.018)
Support for further training: yes (Ref: no)	0.174^{***}	(0.018)
Opportunity for advancement: yes (Ref: no)	0.302^{***}	(0.018)
Atmosphere: collegial (Ref: competitive)	0.303^{***}	(0.018)
Alternative-specific constant: Option B	0.057^{***}	(0.007)
Gender: female (Ref: male)	-0.093^{**}	(0.033)
Family formation: (Ref: early parenthood)	-0.075^{*}	(0.036)
late parenthood	-0.075^{*}	(0.036)
no parenthood	-0.098^{*}	(0.048)
not clear	-0.039	(0.054)
Interaction effect: female X		
Wage: 10% lower	0.001	(0.029)
Wage: 10% higher	-0.010	(0.027)
Workload: 80%	0.069^{**}	(0.022)
Reduction: possible	0.100^{***}	(0.022)
Working hours: flexible	-0.020	(0.022)
Support for further training: yes	0.051^{*}	(0.022)
Opportunity for advancement: yes	-0.050^{*}	(0.022)
Atmosphere: collegial	0.042	(0.022)
Family formation: late parenthood	0.083	(0.050)
Family formation: no parenthood	0.101	(0.064)
Family formation: not clear	-0.047	(0.077)
Interaction effect: family formation: late parenthood X		
Wage: 10% lower	0.012	(0.032)
Wage: 10% higher	-0.032	(0.030)
Workload: 80%	0.013	(0.025)
Reduction: possible	0.065^{**}	(0.024)
Working hours: flexible	0.019	(0.025)
Support for further training: yes	0.036	(0.025)
Opportunity for advancement: yes	0.033	(0.024)
Atmosphere: collegial	-0.003	(0.024)
Interaction effect: family formation: no parenthood X		
Wage: 10% lower	0.020	(0.042)
Wage: 10% higher	-0.054	(0.039)
Workload: 80%	0.044	(0.032)
Reduction: possible	0.032	(0.032)

Table S10 – *Continues on subsequent page*

Table S10 – *Continued from previous page*

Interaction effect: family formation: no parenthood X		
Working hours: flexible	0.040	(0.032)
Support for further training: yes	0.012	(0.032)
Opportunity for advancement: yes	−0.016	(0.032)
Atmosphere: collegial	0.107***	(0.032)
Interaction effect: family formation: not clear X		
Wage: 10% lower	−0.048	(0.048)
Wage: 10% higher	−0.090*	(0.043)
Workload: 80%	0.128***	(0.036)
Reduction: possible	0.009	(0.036)
Working hours: flexible	−0.029	(0.037)
Support for further training: yes	0.044	(0.036)
Opportunity for advancement: yes	−0.025	(0.036)
Atmosphere: collegial	0.031	(0.036)
Interaction effect: female X family formation: late parenthood X		
Wage: 10% lower	0.027	(0.044)
Wage: 10% higher	−0.004	(0.041)
Workload: 80%	−0.007	(0.033)
Reduction: possible	−0.083*	(0.033)
Working hours: flexible	−0.016	(0.033)
Support for further training: yes	−0.073*	(0.033)
Opportunity for advancement: yes	−0.039	(0.033)
Atmosphere: collegial	0.043	(0.033)
Interaction effect: female X family formation: no parenthood X		
Wage: 10% lower	−0.004	(0.057)
Wage: 10% higher	0.065	(0.052)
Workload: 80%	−0.062	(0.043)
Reduction: possible	−0.083	(0.043)
Working hours: flexible	0.021	(0.043)
Support for further training: yes	−0.013	(0.043)
Opportunity for advancement: yes	−0.028	(0.043)
Atmosphere: collegial	−0.080	(0.043)
Interaction effect: female X family formation: not clear X		
Wage: 10% lower	0.078	(0.067)
Wage: 10% higher	0.118	(0.062)
Workload: 80%	−0.060	(0.051)
Reduction: possible	−0.085	(0.051)
Working hours: flexible	0.145**	(0.052)
Support for further training: yes	0.022	(0.051)
Opportunity for advancement: yes	0.015	(0.051)
Atmosphere: collegial	−0.063	(0.051)

Table S10 – *Continues on subsequent page*

Table S10 – *Continued from previous page*

Constant	-0.010	(0.027)
lns1_1_1	-26.932^{***}	(0.320)
lns2_1_1	-28.086^{***}	(0.273)
lnsig_e	-0.874^{***}	(0.006)
N (Choices)	14326	
N (Choice sets)	7193	
N (Respondents)	1796	

Linear probability model nested in participants and choice sets

Standard error in parentheses; * p<0.05, ** p<0.01, *** p<0.001

Table S11: Linear probability model with three-way interaction: Gender Role Attitude X Gender X Job Attribute

Wage: (Ref: as usual)		
10% lower	-0.215^{***}	(0.027)
10% higher	0.033	(0.025)
Workload: 80% (Ref: 100%)	-0.084^{***}	(0.021)
Reduction: possible (Ref: not possible)	0.074^{***}	(0.021)
Working hours: flexible (Ref: fixed)	0.087^{***}	(0.021)
Support for further training: yes (Ref: no)	0.188^{***}	(0.021)
Opportunity for advancement: yes (Ref: no)	0.340^{***}	(0.021)
Atmosphere: collegial (Ref: competitive)	0.321^{***}	(0.021)
Alternative-specific constant: Option B	0.060^{***}	(0.007)
Gender: female (Ref: male)	-0.137^{***}	(0.040)
Gender Role Attitude (Ref: traditional)		
egalitarian	-0.167^{***}	(0.037)
anti-traditional	-0.213^{*}	(0.084)
not clear	-0.167^{***}	(0.049)
Interaction effect: female X		
Wage: 10% lower	0.040	(0.036)
Wage: 10% higher	0.013	(0.033)
Workload: 80%	0.130^{***}	(0.027)
Reduction: possible	0.142^{***}	(0.027)
Working hours: flexible	-0.039	(0.027)
Support for further training: yes	0.052	(0.027)
Opportunity for advancement: yes	-0.099^{***}	(0.027)
Atmosphere: collegial	0.050	(0.027)
Attitude: egalitarian	0.093	(0.049)
Attitude: anti-traditional	0.401^{***}	(0.120)
Attitude: not clear	0.207^{**}	(0.073)
Interaction effect: Attitude: egalitarian X		
Wage: 10% lower	0.055	(0.033)
Wage: 10% higher	0.017	(0.031)

Table S11 – *Continues on subsequent page*

Table S11 – *Continued from previous page*

Interaction effect: Attitude: egalitarian X		
Workload: 80%	0.156***	(0.025)
Reduction: possible	0.097***	(0.025)
Working hours: flexible	0.050*	(0.025)
Support for further training: yes	0.014	(0.025)
Opportunity for advancement: yes	−0.059*	(0.025)
Atmosphere: collegial	0.022	(0.025)
Interaction effect: Attitude: anti-traditional X		
Wage: 10% lower	0.177*	(0.081)
Wage: 10% higher	0.077	(0.073)
Workload: 80%	0.205***	(0.062)
Reduction: possible	−0.078	(0.060)
Working hours: flexible	0.334***	(0.062)
Support for further training: yes	−0.176**	(0.062)
Opportunity for advancement: yes	−0.098	(0.061)
Atmosphere: collegial	0.055	(0.061)
Interaction effect: Attitude: not clear X		
Wage: 10% lower	0.104*	(0.043)
Wage: 10% higher	0.034	(0.039)
Workload: 80%	0.137***	(0.033)
Reduction: possible	0.031	(0.032)
Working hours: flexible	0.104**	(0.033)
Support for further training: yes	0.022	(0.033)
Opportunity for advancement: yes	−0.008	(0.033)
Atmosphere: collegial	−0.053	(0.033)
Interaction effect: female X Attitude: egalitarian X		
Wage: 10% lower	−0.003	(0.044)
Wage: 10% higher	0.005	(0.041)
Workload: 80%	−0.108**	(0.033)
Reduction: possible	−0.132***	(0.033)
Working hours: flexible	0.063	(0.033)
Support for further training: yes	−0.037	(0.033)
Opportunity for advancement: yes	0.064	(0.033)
Atmosphere: collegial	−0.033	(0.033)
Interaction effect: female X Attitude: anti-traditional X		
Wage: 10% lower	−0.285**	(0.109)
Wage: 10% higher	−0.175	(0.100)
Workload: 80%	−0.279***	(0.083)
Reduction: possible	0.009	(0.082)
Working hours: flexible	−0.302***	(0.083)
Support for further training: yes	0.182*	(0.083)
Opportunity for advancement: yes	0.074	(0.083)
Atmosphere: collegial	−0.157	(0.082)

Table S11 – *Continues on subsequent page*

Table S11 – *Continued from previous page*

Interaction effect: female X Attitude: not clear X		
Wage: 10% lower	−0.098	(0.062)
Wage: 10% higher	0.005	(0.058)
Workload: 80%	−0.158**	(0.048)
Reduction: possible	−0.146**	(0.048)
Working hours: flexible	0.017	(0.048)
Support for further training: yes	−0.126**	(0.048)
Opportunity for advancement: yes	0.015	(0.048)
Atmosphere: collegial	0.047	(0.048)
Constant	0.062*	(0.030)
lns1_1_1	−28.263***	(0.266)
lns2_1_1	−30.783***	(0.285)
lnsig_e	−0.878***	(0.006)
N (Choices)	14128	
N (Choice sets)	7064	
N (Respondents)	1766	

Linear probability model nested in participants and choice sets

Standard error in parentheses; * p<0.05, ** p<0.01, *** p<0.001

Table S12. Linear probability model by employment situation

	In employment with secondary education		In employment with tertiary education		In education	
Wage: (Ref: as usual)						
10% lower	−0.188***	(0.020)	−0.168***	(0.038)	−0.116***	(0.025)
10% higher	0.056**	(0.018)	0.026	(0.035)	0.052*	(0.023)
Workload: 80% (Ref: 100%)	−0.003	(0.015)	−0.043	(0.029)	0.111***	(0.019)
Reduction: possible (Ref: not possible)	0.101***	(0.015)	0.186***	(0.029)	0.125***	(0.019)
Working hours: flexible (Ref: fixed)	0.098***	(0.015)	0.125***	(0.029)	0.214***	(0.019)
Support for further training: yes (Ref: no)	0.171***	(0.015)	0.176***	(0.029)	0.234***	(0.019)
Opportunity for advancement: yes (Ref: no)	0.306***	(0.015)	0.297***	(0.029)	0.317***	(0.019)
Atmosphere: collegial (Ref: competitive)	0.331***	(0.015)	0.302***	(0.029)	0.316***	(0.019)
Alternative-specific constant: Option B	0.054***	(0.011)	0.037*	(0.016)	0.067***	(0.013)
Gender: female (Ref: male)	−0.101**	(0.033)	−0.082	(0.050)	0.021	(0.038)
Interaction effect: female X						
Wage: 10% lower	0.050	(0.029)	0.022	(0.044)	−0.018	(0.034)
Wage: 10% higher	−0.008	(0.027)	0.029	(0.041)	0.041	(0.031)
Workload: 80%	0.073***	(0.022)	0.094**	(0.034)	−0.027	(0.026)
Reduction: possible	0.050*	(0.022)	0.001	(0.034)	0.081**	(0.026)
Working hours: flexible	0.084***	(0.022)	−0.061	(0.034)	−0.085**	(0.026)
Support for further training: yes	0.034	(0.022)	0.054	(0.034)	−0.012	(0.026)
Opportunity for advancement: yes	−0.088***	(0.022)	−0.028	(0.034)	−0.074**	(0.026)
Atmosphere: collegial	0.029	(0.022)	0.067*	(0.034)	0.058*	(0.025)
Constant	0.005	(0.023)	0.002	(0.044)	−0.177***	(0.029)
lns1_1_1	−31.092***	(0.428)	−19.612***	(0.627)	−27.034***	(0.607)
lns2_1_1	−29.167***	(0.423)	−19.568***	(0.701)	−28.741***	(0.483)
lnsig_e	−0.857***	(0.009)	−0.872***	(0.012)	−0.905***	(0.011)
N (Choices)	6098		3218		4092	
N (Choice Sets)	1525		806		1024	
N (Respondents)	763		403		512	

Linear probability model nested in participants and choice sets

Standard error in parentheses; * p<0.05, ** p<0.01, *** p<0.001
